# Tribotechnical and Physical Characteristics of a Friction Composite Made of a Polymer Matrix Reinforced with a Complex of Fiber-Dispersed Particles

**DOI:** 10.3390/ma18163847

**Published:** 2025-08-16

**Authors:** Ievgen Byba, Anatolii Minitskyi, Yuriy Sydorenko, Andrii Shysholin, Oleksiy Myronyuk, Maksym Barabash

**Affiliations:** 1 E.O. Paton Education and Research Institute of Materials Science and Welding, National Technical University of Ukraine ‘Igor Sikorsky Kyiv Polytechnic Institute’, 37, Beresteiskyi Ave., UA-03056 Kyiv, Ukraine; byba.evgen@gmail.com (I.B.); aminitsky@gmail.com (A.M.); 2 Institute of Applied Control Systems of the N.A.S of Ukraine, 40, Glushkov Akademika Avenue, UA-03187 Kyiv, Ukraine; 3 Education and Research Institute of Mechanical Engineering, National Technical University of Ukraine ‘Igor Sikorsky Kyiv Polytechnic Institute’, 37, Beresteiskyi Ave., UA-03056 Kyiv, Ukraine; yura_michael@ukr.net; 4 Vice-Rector for International Relations, National Technical University of Ukraine ‘Igor Sikorsky Kyiv Polytechnic Institute’, 37, Beresteiskyi Ave., UA-03056 Kyiv, Ukraine; a.shysholin@kpi.ua; 5 Faculty of Chemical Technology, National Technical University of Ukraine ‘Igor Sikorsky Kyiv Polytechnic Institute’, 37, Beresteiskyi Ave., UA-03056 Kyiv, Ukraine; o.myronyuk@kpi.ua; 6 Technical Center, N.A.S. of Ukraine, 13, Pokrovs’ka Str., UA-04070 Kyiv, Ukraine; 7 Gas Institute of N.A.S. of Ukraine, 39, Degtyarivska Str., UA-03113 Kyiv, Ukraine

**Keywords:** friction disk, wear, graphite, cellulose, phenol formaldehyde, wollastonite

## Abstract

A friction composite material which contains cellulose fiber, carbon fiber, wollastonite, graphite, and resin for use in oil-cooled friction units, hydromechanical boxes, and couplings was developed. The fabrication technique includes the formation of a paper layer based on the mixture of stated fibers via a wet-laid process, impregnation of the layer with phenolic resin, and hot pressing onto a steel carrier. The infrared spectra of the polymeric base (phenolic resin) were studied by solvent extraction. The structural-phase analysis of the obtained material was carried out by the SEM method, and the particle size distribution parameters of the composite components were estimated based on the images of the sample surface. The surface roughness parameters of the samples are as follows: *R*a = 5.7 μm *R*z = 31.4 μm. The tribotechnical characteristics of the material were tested in an oil medium at a load of 5.0 MPa and a rotation mode of 2000 rpm for 180 min in a pair with a steel 45 counterbody. The coefficient of friction of the developed material was 0.11–0.12; the degree of wear was 6.17 × 10^−6^ μm/mm. The degree of compression deformation of the composite is 0.36%, and the compressive strength is 7.8 MPa. The calculated kinetic energy absorbed and power level are 205 J/cm^2^ and 110 W/cm^2^, respectively. The main tribotechnical characteristics of the developed friction material correspond to industrial analogues.

## 1. Introduction

One of the most promising areas of materials science and powder metallurgy is the production of sintered friction polymeric materials. Friction materials are widely used in transmission and braking units and mechanisms of the automotive industry, special-purpose equipment, machine tools, machines, and mechanisms [1,2,3]. The development of friction materials for liquid transmissions has evolved significantly, transitioning from early use of purely metallic components to more advanced composite systems to meet increasing demands for durability and performance. Initial designs prioritized mechanical strength and thermal stability, but often lacked sufficient compatibility with transmission fluids and smooth engagement characteristics. This progression ultimately led to the widespread adoption of paper-based friction materials, which have a developed pore [4]. These materials ensure the required value of the dynamic and static friction coefficients, high wear resistance, effective wear resistance, and high thermal conductivity [5,6,7]. As it is shown in [8,9,10,11], the final properties of material can be flexibly adjusted via modification of the pore structure, size, and the mechanical properties (strength/elasticity of their walls. The addition of particles of different nature (cellulose, carbon fibers, finely dispersed particles of wollastonite, graphite, palm kernel shell) to a polymer matrix made of phenol-formaldehyde resin improves the physical, mechanical, and tribotechnical properties of friction materials [12,13,14]. In particular, a reinforcing carbon phase such as graphite or graphene in structures with a metal matrix can improve performance under friction and wear conditions. Graphite or graphene is used in tribotechnical materials as a solid lubricant to reduce wear on the surfaces of parts during friction [15,16,17]. This is due to the formation of a thin graphite film that protects the surface from wear through the lubricating effect.

Paper-based friction materials are widely used in automatic transmissions, wet clutches, and braking systems, where controlled friction under lubricated conditions is essential. These materials ensure consistent torque transmission [18], precise shifting, and effective braking performance [19] across a range of automotive, industrial, and heavy-duty machinery applications. Their ability to maintain friction stability, minimize noise and vibration, and withstand thermal and mechanical stresses makes them a critical component in the design of high-performance powertrain and hydraulic systems [20].

The authors determined the effect of adding fibers and solids that are mixed in the paper process, while drying, compacting and forming a paper-like material [21]. The paper material is then impregnated with a resin solution, dried, and cured under a press. The proposed technology for the production of friction materials belongs to the “paper” type, when the tribotechnical characteristics of the product are improved by strengthening the material with fibers and particles. The analysis of literature sources shows that there is no information on the use of fiber additives of different nature to modify friction materials.

The study aimed to search for the optimal, economically feasible chemical composition (formulation) and manufacturing technology of friction materials and determine their tribotechnical properties.

## 2. Methods and Experimental Techniques

An effective technology for manufacturing paper friction materials with appropriate tribotechnical characteristics under lubrication conditions, which was used for sample fabrication, is described in [22].

The following components were used as starting materials:

- cellulose fibers with a fiber length of 1.5–5 mm;
- carbon fiber—Carbiso chopped carbon fiber (Carbiso CT, ELG CARBON FIBRE Ltd., Bliston, UK) 3 mm long;
- micronized woolastonite of W200, Nordkalk, Finland (friction particles);
- flake-like grade GS-4, Zavalivskiy Graphite LLC, Zavallya, Ukraine;
- phenol-formaldehyde resin of the Modofen OD, Ciech S.A., Poland, Nova Sarzyna.

The friction composition formulation used in the study (Table 1) is the result of preliminary testing of an average formulation of paper friction materials [US 2010/0330335 A1 2010, US 2010/0256259 A1, US 2022/0243779 A1 2022] for liquid systems. The essence of the preliminary experiment was that the content of wollastonite, graphite, and phenol-formaldehyde resin varied at two levels to achieve the target kinetic friction coefficient of 0.11 and decrease the degree of wear to values lower than 18 × 10^−6^ μm/mm.

The resin content variation is performed by the use of a series of isopropanol solutions for impregnation. After the production cycle, the actual resin content is determined, and the optimal solution concentration selected. This approach is caused by the fact that each formula has its own specific absorption capacity that is determined by the integral interaction of the structure parameters of the friction layer base, such as porosity, filler particle morphology, fiber content, and arrangement.

**Figure 1:**
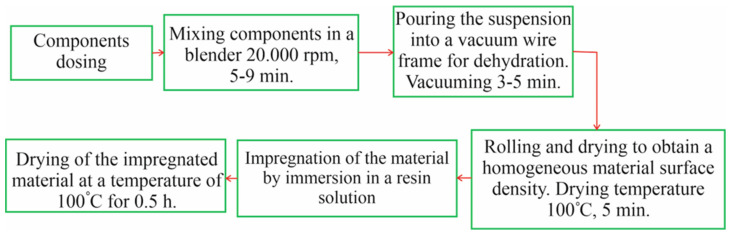
Block diagram of a typical technological process of producing friction material.

Figure 1 shows a flowchart of the friction material manufacturing process. The main stages of this process are dosing of components, mixing them in a blender, stabilization of the suspension and water separation, squeezing, drying, and subsequent impregnation with resin (Figure 1). The overall performance of friction materials depends on the various components and their ability to convert kinetic energy into heat through friction. Therefore, the material properties of the lining material are of great importance, including wear, friction coefficient, and mechanical behavior. Typically, phenol-formaldehyde, some fibrous materials, and carbon fibers are preferred in appropriate proportions [20]. The friction material produced according to this scheme is widely used in automatic transmissions (AT) of heavy-duty vehicles [21,23].

The technology for manufacturing friction materials involves weighing the system components with an accuracy of 0.01 g. Cellulose and carbon fibers were loaded into a blender container and filled with hot water in the ratio of 4 g of dry matter to 200 g of water. The water temperature was 80–100 °C. The grinding process lasted 3–5 min at 20,000 rpm. Next, wollastonite and graphite were loaded into the container and additional dispersion was carried out for 2–4 min at 20,000 rpm. A total of 80 mL of the resulting pulp was taken and loaded into a Buechner funnel with a pre-installed metal and paper filter. This approach ensures the retention of pulp fibers and complete evacuation of water. The vacuuming process continued until the water was completely removed so that the material would not be destroyed during its movement (3–5 min). Next, the resulting disk was placed on a cloth and rolled through rollers to further remove water and obtain a sample of uniform thickness (2.35–2.55 mm). The resulting material was dried at 100 °C under low pressure to completely remove moisture and avoid deformation. Impregnation with phenol-formaldehyde was performed at an excess of resin. The container with the resin solution and the friction material was placed in a vacuum chamber and kept for 0.5–1 min in a vacuum with a pressure of 0.95–1 atm to completely remove air from the pores. After vacuuming, the material was rolled through rollers to remove excess resin. Drying took place for 30 min at a temperature of 100 °C without pressing. As a result, the obtained friction material had a thickness of 1.85–2.00 mm.

The mineral composition of the samples was studied by X-ray diffraction [24] using an Ultima-IV diffractometer manufactured by Rigaku, Tokyo, Japan (Kyiv, National Technical University of Ukraine “Igor Sikorsky Kyiv Polytechnic Institute”). The diffracted radiation was recorded on a PC. The identification of peaks on the diffractograms was performed using the ICDD PDF-2 and PDF-4 software package. Chemical analysis of the samples was performed by X-ray fluorescence analysis using an Expert 3L instrument (LLC SPE Institute of Analytical Control Methods, Kyiv, Ukraine).

In order to study the structural features of the obtained friction material, the infrared spectra of samples 1–5 (Table 2) were measured using Spekord IR-75 (Carl Zeiss AG, Oberkochen, Germany) and Nicolet 4700 equipment (ThermoFisher Scientific, Waltham, MA, USA). The tribotechnical characteristics of the samples, determination of static compressive strength, degree of wear, and average coefficients of kinetic and static friction were tested on an automated tribological complex (ATC) under quasi-static and cyclic conditions in the transmission oil environment. The M-22 PV friction machine was used to determine the kinetic friction coefficient, power level, and absorbed kinetic energy, which provides testing under radial and end loading schemes, and also allows changing of the friction torque and total linear wear of the samples during testing. Thermogravimetric analysis was performed using a Derivatograph Q-1500 D systems Paulic, Paulic-Erday, Budapest, Hungary. The following parameters were used for the TGA analysis: heating rate—5 C/min, final temperature—1100 °C, environment—air, holding time—300 s, sample weight—56.3 mg. To determine the surface roughness, a micron alpha profilometer was used, and the values of the geometric parameters of the disk were measured by a module based on the 296 profilometer, according to ISO 21920-2:2021 [25]. The module allows measuring profiles up to 40 mm long and 1 mm high with an accuracy of 1 μm, using a diamond needle with a rounding radius of 0.01 mm and the use of an additional mathematical apparatus. The kinetic coefficient of friction and the degree of wear of friction materials in the transmission oil medium at a load of 5 MPa and a speed of 2000 rpm were determined on an experimental setup with a strain gauge system. During the tests, the temperature at the point of contact of the friction pair was recorded, since this parameter is responsible for the value of the absorbed kinetic energy.

The surface profile, roughness, and tribotechnical characteristics of the obtained friction composite are shown in comparison with the industrial sample RE294017 of the John Deere (Moline, IL, USA) friction clutch disk.

The microstructure of the friction material was studied by light (FMA050, AM-Scope Ltd., Irvine, CA, USA) and scanning (SEM) microscopy (Mira3 LMU, Tescan JSC, Brno, Czech Republic). The device is equipped with an attachment for energy dispersive microanalysis. INCA Energy software Version 4.15 was used for data processing and quantitative microanalysis according to the scheme of correction of the XRD matrix effects. This approach, based on the PhiRho-Z method, ensures high calculation accuracy and precise analysis of elements in the sample matrix.

## 3. Results and Discussions

**Figure 2:**
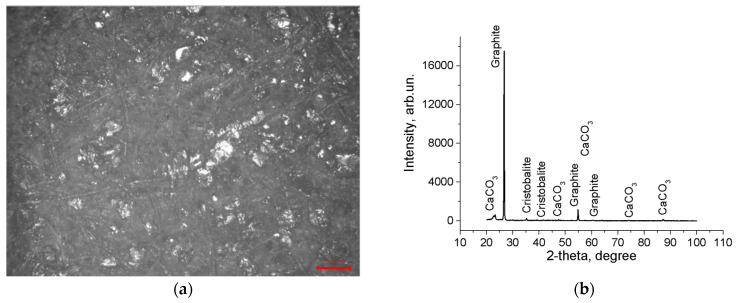

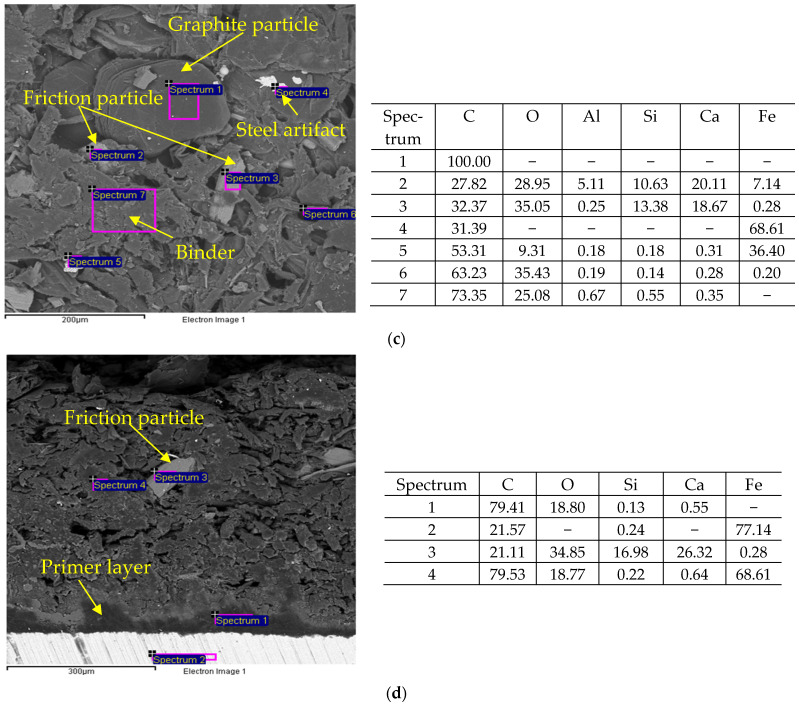
Microstructure, phase composition and EDS analysis of the formed friction material: (**a**)—fiber morphology with inclusion particles, optical microscopy, (**b**)—phase composition diffractogram, (**c**)—surface; (**d**)—cross section.

The results of phase and elemental analysis of the obtained friction material are shown in Figure 2a–d. The data obtained indicate that the fibers have a complex chemical composition, mainly in the form of oxides. The obtained friction material, according to the results of X-ray analysis, consists of a cellulose base, graphite, cristobalite SiO_2_, and calcium carbonate CaCO_3_ (Figure 2b). The chemical composition of the sample elements was studied using the EDS technique (Figure 2c,d). The large particle (spectrum 1) has a diameter of 200 μm and consists entirely of carbon. Given its layered structure, it can be assumed that it is graphite. The absence of oxygen in this case indicates that the surface of this particle is not covered by a bonding film. The crystalline particles (spectrum 2 of Figure 2d) contain C, O, Si, and Ca atoms, which can be attributed to such minerals as CaSiO_3_, SiO_2_, or CaCO_3_. The SEM images contain particles containing iron (spectrum 4, Figure 2c,d), which are particles of the steel base formed during the sample preparation. The bulk of the material (spectra 6 and 7) is a carbon–oxygen binder—the cured resin.

The structure of the resulting friction material is porous and consists of fibers and relatively coarse particles in a polymer matrix (Figure 2a). The sizes of pores and graphite particles were estimated using AMIC software v.1.0 based on image analysis (Table 2).

In cross-section, there are no coarse graphite particles, which is explained by the use of lamellar varieties of this additive to prevent adhesion of friction pairs. In contrast, the mineral filler particles (spectrum 3, Figure 2c,d) are located throughout the thickness of the friction material layer, with lower porosity in the lower part of the layer. The developed friction material is attached to the surface of the steel bearing base using an adhesive (primer, spectrum 1, Figure 2d). The elemental composition of this layer is really close to the composition of the material’s binder (compared to spectrum 4, Figure 2d).

On the microstructures of Figure 2a,c,d, one can see the fiber filler, dispersed additives, and polymer binder particles. The presence of a porous structure in a friction material is important because during operation, it is the pores that ensure the circulation of oil in the material, which is both a coolant and a lubricant [26].

**Figure 3:**
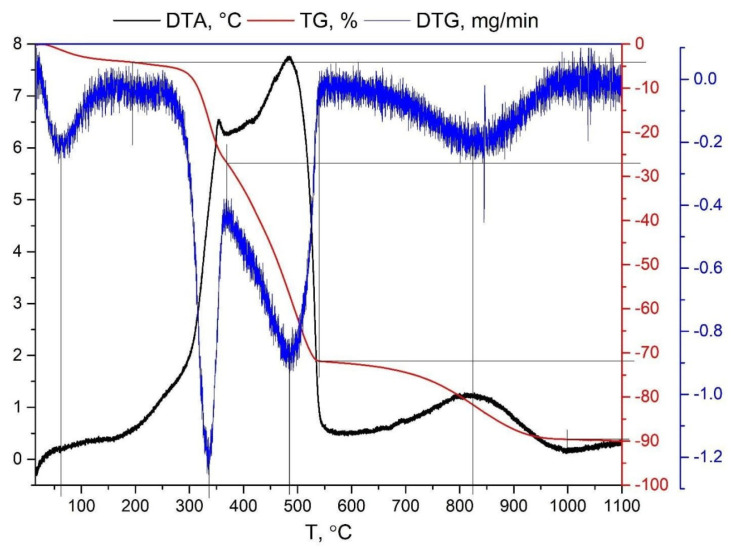
TGA-DTA curve of the friction material.

The TGA analysis (Figure 3) indicates the presence of a low-temperature stable ingredient (cellulose fibers) with a peak at 336 °C and a mass loss of 20.8 wt.%. Phenolic resin carbonizes with a peak at 485 °C and a mass loss of 45.6 wt.%, which is close to the behavior of new varnish resin (not modified with siloxane or imide fractions). The graphite destruction temperature is 825 °C, and the mass loss in this case is 18 wt.%. The residual content (10 wt.%) of the products of thermal degradation of the composite in air is a crystalline solid (friction particles).

The mineral composition of the sample (Figure 2b, determined by XRD analysis and interpreted automatically using the pdf library) shows that the graphite content is 34%, cristobalite—18.3%, CaCO_3_—1.1%.

**Figure 4:**
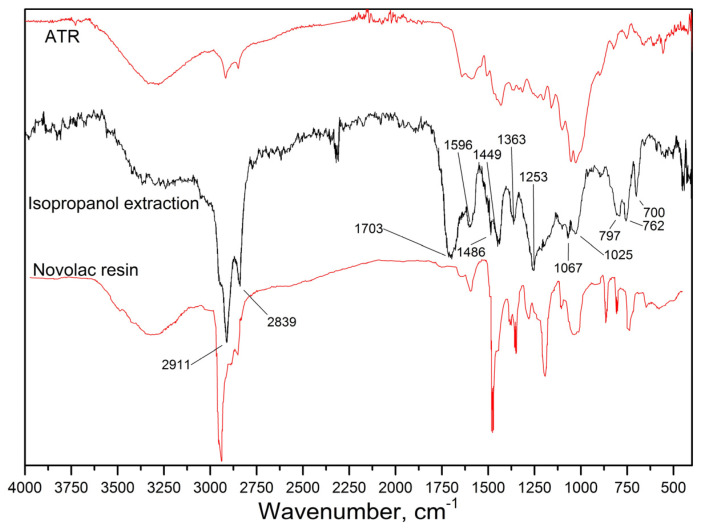
Comparison of the experimental spectra (ATR and isopropanol extract) with the reference spectrum (novolac resin).

The resin part of the composite was studied by solvent extraction. Despite the insolubility of the material in most solvents (aliphatic and aromatic hydrocarbons, ethers, alcohols), it was found that extraction occurs under boiling isopropanol conditions. The infrared spectrum of the isopropanol extract in the range of 4000–400 cm^−1^ in the transmission mode was obtained (Figure 4. Extraction with isopropanol). Along with this, the overall surface spectrum of the friction composite was characterized by ATR-FTIR in the same spectral range (Figure 4. ATR).

**Figure 5:**
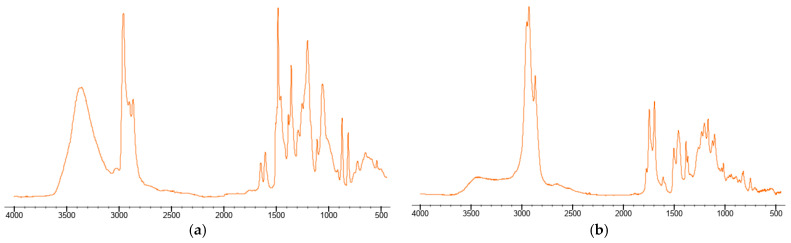
Infrared spectra of: (**a**)—phenolic resin modified with alkyl, (**b**)—rosin modified phenolic resin.

At 1595 cm^−1^, broad bands appear corresponding to the vibrations of the aromatic C=C double bond in the phenyl group (Figure 5a). In the region of 2950–2800 cm^−1^, two intense bands at 2911 and 2839 cm^−1^ are observed, which are due to the stretching of -CH_2_ groups of aromatic and aliphatic structures at the base of the chain. The absorption peak at 1449 and 1363 cm^−1^ -CH_2_ bending. A significant peak at 3300 cm^−1^ corresponds to -OH tensile vibrations. The peak at 1704 cm^−1^ corresponds to the C=O stretching of ketone groups. The C-O stretching of the alkylaryl ether corresponds to the peak at 1253 cm^−1^.

The data obtained indicate that the extract obtained is similar to the phenolic resin of novolac, but the resin used in the friction material is modified (Figure 5b). The spectrum shows that the resin was modified with a substance that has C=O groups in its structure. The modifier or additive is easier to extract than the gel components of the resin. The ATR spectrum confirms the presence of Si-O bonds in the structure of the material, which is most likely a filler—CaSiO_3_ or SiO_2_.

The friction material contains cellulose fibers, which is proven by the coincidence of its FTIR spectrum with reference [27].

**Figure 6:**
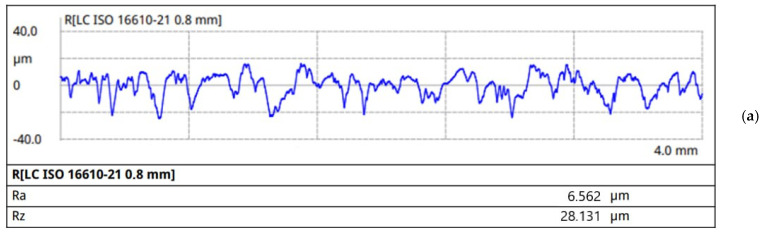

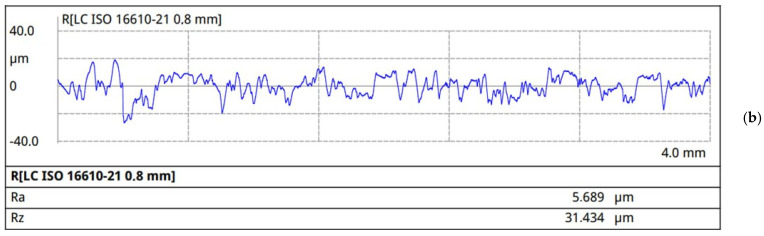
Surface profiles of samples: (**a**)—industrial sample, (**b**)—friction material.

Given that one of the main characteristics that affects the friction process is the surface roughness of the material, the roughness of the samples was determined using a profilometer. The surface roughness of the experimental friction material is *R*a = 5.689 µm; the surface profilogram is shown in Figure 6a,b. The measurement results indicate that the average height of profile irregularities is 4.0–7.5 μm, with individual deviations up to 18.0–20.0 μm.

According to the results of measurements of the surface roughness of the samples, the prototype sample is similar to the industrial sample in terms of the arithmetic mean deviation of the profile *R*a and the greatest height of the profile *R*z. At least 10 measurements were made per sample, with a relative deviation of 5%. The data of roughness measurements of the samples from the developed friction material and the industrial one are given in Table 3.

The coefficient of friction and the degree of wear of friction material samples were determined. After the tests, the depth of wear was determined on the module assembled on the basis of a model 296 profilometer.

**Figure 7:**
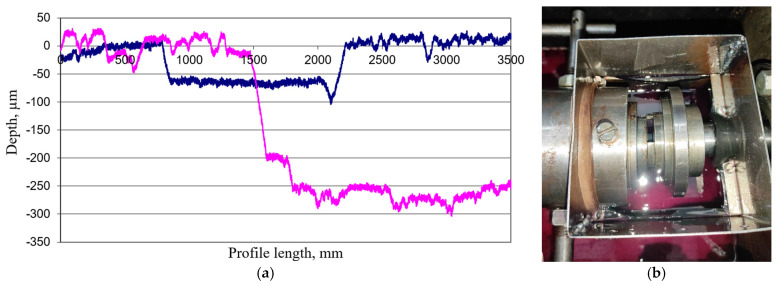
Results of tribotechnical tests of samples: (**a**)—wear depth, blue—developed friction material sample, pink—industrial sample (**b**)—assembly scheme of the friction unit in oil medium.

The kinetic coefficient of friction and the degree of wear of friction materials were determined on an experimental setup with a strain gauge system (Figure 7a,b). To ensure the correctness of the results, five tests were conducted for each sample, with an average statistical deviation of 5–7%. The test results are given in Table 4. To determine the performance characteristics of the obtained material, the wear depth was determined using a profilometer, and the wear intensity was calculated according to the obtained geometric parameters.

According to the research results, the optimal chemical composition and technological parameters of the friction material were determined and its technical parameters were determined (Table 5).

To determine the degree of deformation, the Archard formula was used, which takes into account the degree of wear and hardness of the material [28].

$$w=k\frac{N\cdot L}{H}$$

where *w*—wear, mm^3^, *k*—deformation coefficient (degree), *N*—normal force, N, *L*—sliding path, mm, *H*—hardness of the wearing material, MPa (N/mm^2^), *P*—load, MPa.

Having the information about the amount of linear wear, load, and material properties, one can determine the strain coefficient using the formula:

$$k=\frac{h\cdot H}{P\cdot L}$$

Taking into account the values of the known quantities,

For the original sample,

$$k=\frac{0.284\;\mathrm{mm}\cdot 490\;\mathrm{MPa}}{5\;\mathrm{MPa}\cdot 844\;\mathrm{mm}}=0.0033$$

For experimental sample,

$$k=\frac{0.315\;\mathrm{mm}\cdot 490\;\mathrm{MPa}}{5\;\mathrm{MPa}\cdot 861.3\;\mathrm{mm}}=0.0036$$

The results of comparing the technical characteristics of friction materials showed that the developed material corresponds to the standard in terms of the main parameters, namely the kinetic and static coefficient of friction and the degree of wear. Differences in the characteristics of the kinetic energy absorption and power level are due to the fact that these values were not determined directly, but were calculated using the Archard formula, which takes into account the scale factor.

## 4. Conclusions

A friction paper composite that includes cellulose fiber, carbon fiber, wollastonite, graphite, and phenolic resin was manufactured and characterized. A respective fabrication process that starts from making the paper material by wet-laid process, its pressing and drying, impregnation with the phenolic resin isopropanol solution followed by pressing, drying, and formation of friction layer on steel substrate is described. The obtained material layer has a uniform distribution of components in the polymer matrix, has well-developed porous structure that provides oil circulation in the material for cooling and lubrication.

The surface roughness of the developed friction material is *R*a = 5.689 μm, and the average height of profile irregularities is 4.0–7.5 μm, with individual deviations up to 18.0–20.0 μm. A set of comparative tests of the developed materials with an industrial analog of the gearbox friction clutch disk was performed. It was found that the developed friction material is more stable during wear tests at elevated temperatures. It is shown that the wear depth of the experimental friction material is 2.5 times lower than that of the industrial one. The thermal stability of material is proven by thermogravimetry, which shows that the first significant weight loss effect for the material happens at 485 °C and has a magnitude of 45.6% wt., which is close to the behavior of phenolic novolac resin.

It was determined that the friction coefficients of both materials are the same and correspond to values of 0.12. The degree of wear of the developed friction material is significantly lower than that of the reference material (16.8–10^−6^ μm/mm) and is 0.52∙10^−6^ μm/mm. The degree of deformation of the developed sample under compression is 0.35%. The compressive strength of the manufactured material exceeds that of its industrial counterpart and is 7.8 MPa. The calculated kinetic energy absorption and power level are 205 J/cm^2^ and 110 W/cm^2^.

The developed friction paper composite demonstrates a combination of thermal stability, mechanical strength, and wear resistance that makes it a promising candidate for use in high-performance frictional components operating under elevated thermal and mechanical loads. Fields of application include automotive and aerospace transmissions, particularly in wet clutch systems where oil circulation is essential for cooling and lubrication. The porous microstructure, which enables efficient lubricant flow, along with the material’s high compressive strength and low wear rate, also renders it suitable for advanced braking systems, industrial drive units, and mechatronic actuators in robotics.

Moreover, the compatibility of the fabrication method with scalable wet-laid processing techniques enhances its applicability in the production of eco-efficient friction elements for gearboxes and automated machinery requiring stable performance over prolonged service periods. Future developments of these results may focus on the optimization of pore architecture to further tailor tribological and thermal properties, as well as on the integration of alternative polymer matrices or functional fillers to expand the material’s performance envelope.

## Figures and Tables

**Table 1:** Composition of the paper friction material.

Component	Quantity, wt.%
Cellulose fiber	23.5
Carbon fiber	10.6
Wolastonite	12.9
Graphite	11.7
Resin	41.3
**Total**	**100.0**

**Table 2:** Calculations of the structural components of the sample on the surface.

Structure Element	Fraction by Area, %	*D*_min_, µm	*D*_max_, µm
Pores	52.11	25.14	280.36
Graphite	28.58	11.34	240.12
Fibers	19.31	75.51	640.15

**Table 3:** Characteristics of the surface roughness of friction material samples (arithmetic mean deviation of the profile *R*a and the greatest height of the profile *R*z).

Number of Sample	*R*a, μm	*R*z, μm
industrial	6562	28,131
friction material	5689	31,434

**Table 4:** Depth of wear and degree of wear of friction material samples.

Sample	Wear Depth, μm	Degree of Wear, μm/mm	Kinetic Friction Coefficient
Industrial	222.17	16.8·10^−6^	0.11
Experimental	81.67	6.17·10^−6^	0.11

**Table 5:** Technical parameters of the developed friction material and the reference.

Characteristic	Experimental Sample	Industrial Sample
Static compressive strength	7.8 MPa	6.9 MPa
Degree of deformation during compression	0.36%	0.33%
Kinetic friction coefficient (at 5.0 MPa and 2000 rpm)	0.11	0.11
Degree of wear and tear	6.17∙10^−6^ μm/mm	16.8∙10^−6^ μm/mm
Kinetic energy absorbed	205 J/cm^2^	212 J/cm^2^
Power level	110 W/cm^2^	120 W/cm^2^
Average static friction coefficient	0.12	0.12

## Data Availability

The original contributions presented in this study are included in the article. Further inquiries can be directed to the corresponding author.
